# Integrating Temperature-Dependent Life Table Data into a Matrix Projection Model for *Drosophila suzukii* Population Estimation

**DOI:** 10.1371/journal.pone.0106909

**Published:** 2014-09-05

**Authors:** Nik G. Wiman, Vaughn M. Walton, Daniel T. Dalton, Gianfranco Anfora, Hannah J. Burrack, Joanna C. Chiu, Kent M. Daane, Alberto Grassi, Betsey Miller, Samantha Tochen, Xingeng Wang, Claudio Ioriatti

**Affiliations:** 1 Department of Horticulture, Oregon State University, Corvallis, Oregon, United States of America; 2 Research and Innovation Centre and Technology Transfer Centre, Fondazione Edmund Mach, San Michele all’Adige, Trento, Italy; 3 Department of Entomology, North Carolina State University, Raleigh, North Carolina, United States of America; 4 Department of Entomology and Nematology, University of California Davis, Davis, California, United States of America; 5 Department of Environmental Science, Policy & Management, University of California, Berkeley, California, United States of America; International Atomic Energy Agency, Austria

## Abstract

Temperature-dependent fecundity and survival data was integrated into a matrix population model to describe relative *Drosophila suzukii* Matsumura (Diptera: Drosophilidae) population increase and age structure based on environmental conditions. This novel modification of the classic Leslie matrix population model is presented as a way to examine how insect populations interact with the environment, and has application as a predictor of population density. For *D. suzukii*, we examined model implications for pest pressure on crops. As case studies, we examined model predictions in three small fruit production regions in the United States (US) and one in Italy. These production regions have distinctly different climates. In general, patterns of adult *D. suzukii* trap activity broadly mimicked seasonal population levels predicted by the model using only temperature data. Age structure of estimated populations suggest that trap and fruit infestation data are of limited value and are insufficient for model validation. Thus, we suggest alternative experiments for validation. The model is advantageous in that it provides stage-specific population estimation, which can potentially guide management strategies and provide unique opportunities to simulate stage-specific management effects such as insecticide applications or the effect of biological control on a specific life-stage. The two factors that drive initiation of the model are suitable temperatures (biofix) and availability of a suitable host medium (fruit). Although there are many factors affecting population dynamics of *D. suzukii* in the field, temperature-dependent survival and reproduction are believed to be the main drivers for *D. suzukii* populations.

## Introduction

*Drosophila suzukii* Matsumura (Diptera: Drosophilidae) is an economic pest of small and stone fruit in major production areas including North America, Asia and Europe [1]–[6]. Female *D. suzukii* oviposit into suitable ripening fruits using a serrated ovipositor [5], [6]. This is unique compared to other drosophilids, including the common fruit fly, *D. melanogaster*, which oviposit into overripe or previously damaged fruit [7], [8]. Developing fruit fly larvae render infested fruit unmarketable for fresh consumption and may reduce processed fruit quality and cause downgrading or rejection at processing facilities. In Western US production areas, *D. suzukii* damage may cause up to $500 million in annual losses assuming 30% damage levels [2], and $207 million in Eastern US production regions [9]. Worldwide, the potential economic impacts of this pest are staggering.

Pesticide applications have been the primary control tactic against *D. suzukii* both in North America and in Europe. The most effective materials are those that target gravid females, including pyrethoids, carbamates, and spinosyns [1], [10], [11]. These applications are timed to prevent oviposition in susceptible ripening host crops [10], [11]. In the Pacific Northwest, many growers have adopted scheduled spray intervals of 4–7 days [10], [11]. This prophylactic use of insecticide is unsustainable as growers have a limited selection of products and modes of action. This could ultimately lead to *D. suzukii* becoming resistant and may cause secondary pest problems because of negative effects on beneficial organisms. Furthermore, production costs have increased substantially in crops where *D. suzukii* must be managed [6].

Effective sampling methodology for *D. suzukii* is lacking despite extensive efforts to improve trap technology or determine effective fruit infestation sampling protocols. Theoretically, traps to capture adult flies should aid growers in the timing of spray applications so that insecticides could be used more judiciously. Traps baited with apple cider vinegar or a combination of sugar-water and yeast are currently used to monitor adult *D. suzukii* flight patterns [12]–[14]. However, without standard methods for trapping or management thresholds based on trap count data, it is questionable how much is gained by establishing and monitoring traps in crops. Establishing, monitoring, and maintaining traps is very labor intensive and the costs do not justify the benefits for many growers. Historically, trap data has not provided a reliable warning against *D. suzukii* attack, especially for susceptible crops in high-density population areas where considerable oviposition can occur in short time periods [5], [14]. Currently, no significant differences are found in any traps used for monitoring *D. suzukii* given differences between crops and environments where traps have been tested [14]. Monitoring fruit infestation levels to guide management may also be impractical. It is unclear how many samples would be needed to accurately determine infestation levels. Furthermore, by the time larvae are detected in the fruit, it is too late for management action and damage has already occurred. No detailed studies could be found using monitoring for fruit infestation for this pest, and precision of sampling methodology is currently unavailable.

Degree-day (DD), or phenology models, are standard tools for integrated pest management in temperate regions and are used to predict the life stages of pests in order to time management activities and increase the effectiveness of control measures. Degree-day models work best for pests with a high level of synchronicity and few generations [15]–[19]. Our data suggest that *D. suzukii* has short generation times, high reproductive levels, and high generational overlap compared to other dipteran fruit pests [8], [15], [17], [19]. Given this life history, stage-specific population models represent an alternative and potentially more applicable tool for modeling pest pressure. Pest population estimates may be greatly improved by employing additional tools such as mark-recapture [20] and analytical or individual-based models [21]–[23]. The ability to describe and forecast damaging pest populations is highly advantageous for fruit producers, policy makers, and stakeholder groups [20], [21], [24]–[27]. Many such studies have been directed at forecasting populations of medically important insect species [28], [29]. The major factors affecting survival, fecundity and population dynamics of drosophilids include temperature, humidity, and the availability of essential food resources [30]–[32]. Therefore, an improved understanding of the role of temperature on *D. suzukii* may provide for a better understanding its seasonal population dynamics.

In this paper, we present a population model for *D. suzukii* that represents a novel modification of the classic Leslie projection matrix [33], [34], which has proven to be one of the most useful age-structured population models in ecology, with applications for diverse organisms including plants, animals, and diseases [35]–[38]. Our modification accounts for the effect of temperature on the survival and fertility of *D. suzukii* in calculating population growth of the organism. Typically, researchers have introduced elements of environmental stochasticity to matrix models to study environmental effects on population trajectories [38]. However, our approach relies on temperature-dependent estimations of age-specific fecundities and survival that are determined by models fit to life table data generated for multiple temperatures [8]. Our environmentally dependent matrix model is unique in that it does not rely on simulation of environmental effects on populations, but the matrix itself is recalculated at each iteration in direct response to environmental input. Model predictions were run under environmental conditions from different regions to illustrate variation between and within study sites in different years. These simulations make important predictions about age structure and population trends that have implications for pest management both in a broad sense and with regional specificity. This modeling tool may improve current management practices by predicting pest pressure independent of trap catches or samples of infested fruit. We also see potential applications of this model for research in other fields of study and for broadening the understanding of how pests interact with the environment.

## Materials and Methods

### Study sites

We selected three US regions with distinct climatic differences as test cases for comparison to examine model predictions. These sites were: Salem, Oregon; Wilmington, North Carolina; and Parlier, California. To examine the effects of environmental influences within a single region, we compared Pergine, Riva del Garda and Sant’Orsola, in Trentino Province of Northern Italy, for the 2013 season. For annual climatic variation, we compared model predictions based on environmental data from a site near Salem (Oregon, US) and Pergine (Trentino, Italy) in 2012 and 2013.

### Temperature data

Daily mean temperature data were acquired from four fruit production regions from weather stations that were proximal to the field sites where trap and fruit infestation data were procured: Salem Oregon, US (44°43′N, 123°02′W, 70 m elev., years 2012–2013 [39], Parlier, California, US (36°36′N; 119°30W, 110 m elev., year 2013) [40], Wilmington, North Carolina, US (34°13′N; 77°56W, 3 m elev., year 2013) [41] and Trentino, Italy (years 2012–2013; C. Ioriatti, pers. comm.). The US weather data were used to estimate the impact of whole-season climate differences for 2013 on *D. suzukii* population numbers. Temperature data from Trentino during 2013 were used to estimate the role of temperature as affected by elevation and moderating water bodies, *i.e.*, Trentino locations of Riva del Garda (46°00′N; 11°13E, elev. 60 m), Pergine (46°06′N; 11°14′E, elev. 500 m) and Sant’Orsola (46°06′N; 11°17′E, elev. 1150 m) on *D. suzukii* population dynamics. Temperature data from 2012 and 2013 representing Salem and Pergine were used to illustrate annual climatic variation effect on predicted populations of *D. suzukii*.

### Population and stage-specific estimation

The population projection model was written in the open-source statistical environment R version 3.0.2 [42]. The model calculated the matrix based on mean temperature input. Briefly, the matrix (*M*) calculations were based on age-specific regressions of temperature-dependent population parameters as highlighted by Tochen et al. [8]. Whereas immature life stages of *D. suzukii* may experience different environmental conditions than adults because these life stages are completed within the fruit, in this study, ambient air temperatures were used to predict population dynamics for all life stages. To return age-specific population vectors for 50 age-classes of *D. suzukii* for each test case, a vector of mean daily temperatures for each site was input into the R statistical interface. The biofix, or the point where the model began in the spring, was determined using methods described in Tochen et al. [8]. Biofix essentially described the earliest point in the season when the temperature allows the population to increase. Calculations for population estimates were initiated on the biofix date of 2 February in Parlier and 1 April in Wilmington (2013) and Salem (2012 and 2013). In Pergine and Sant’Orsola, estimates were initiated on 6 April (2012 and 2013). The population matrices were initiated with 100 flies in the population vector (*n*) for 41–50 day-old females (*n*_41_…*n*_50_) based on the assumption that females of this age group represent flies that would be emerging from diapause in spring [43]. The log-transformed sum of *D. suzukii* from all life stages (vector sum) for each day represented the total population estimate except where age distributions are considered. For daily age distribution of *D. suzukii* from Parlier, Salem and Wilmington during 2013, 1–3 day-old *D. suzukii* were classified as eggs, 4–7 day-olds were larvae, 8–9 day-olds were pupae, and 10–50 day-olds were classified as adults [8]. Among the most important assumptions of the model are that populations of *D. suzukii* would not be limited by host availability, are not density dependent, do not exhibit Allee effects, and that response to current temperature is not dependent on previous temperature exposure.

### *Drosophila suzukii* trap counts and fruit infestation

Seasonal weekly trap catches of *D. suzukii* were recorded in all study sites, except Riva del Garda, but model estimations for this location was included because the climate here is much different from the other locations studied in Italy. Trap counts were pooled data from commercial blueberry fields in Wilmington (28 traps); unsprayed apricot, blackberry, blueberry, cherry, peach, and citrus orchards in Parlier (18 traps); commercial blueberry fields and surrounding blackberry vegetation in Salem (50 traps); strawberry, blackberry, cherry and blueberry fields in Pergine (6 traps); and unsprayed strawberry and raspberry fields in Sant’Orsola (6 traps). In Wilmington and Salem, traps were made of clear plastic cups, ca. 1 liter in volume each. Each trap had 6–15 entrance holes 4.5–9 mm in diameter [14]. Trap baits in Wilmington consisted of a yeast and sugar water mixture containing 6 g yeast and 40 g sugar dissolved in 710 ml water. In Salem, traps were baited with 100–200 ml natural apple cider vinegar (H.J. Heinz Company, L.P.) and 1–3 ml unscented liquid soap to break water surface tension. In Parlier the traps were made to the specifications of the “Haviland Trap” design for *D. suzukii* monitoring [14]. A 750-ml plastic container (Newell Rubbermaid, Atlanta, GA) served as the basin for each trap. A 7.5-cm diameter hole was cut in the lid, over which a piece of 0.6-cm wire mesh was attached. Each trap was covered with a Pherocon trap cover (Trécé Inc. Adair, Oklahoma), which had a built-in wire hanger. Each trap was filled with 250–300 ml of apple cider vinegar (Great Value Apple Cider Vinegar, Wal-Mart Stores, Inc., Bentonville, AR) with 15 ml of unscented soap (Bon-Ami Company, Kansas City, MO) added as a surfactant to each container (3.78 liters) of vinegar. In Trentino the containers were 1000-ml graduated white polyethylene bottles (Kartell) filled with 200 ml apple cider vinegar (Prantil, Via della Bonifica, 8 38010 Priò di Vervò, Val di Non, Trentino). All traps were placed near the fruiting level of host plants or on stable surfaces in shaded areas and were checked weekly. The contents (vinegar and specimens) of each trap were collected into a separate container that was taken to the laboratory for processing, and at the same time, the traps were refilled with fresh apple cider vinegar and unscented soap, as described above, in the field. The liquid and contents from each trap sample were strained in the laboratory and the numbers of adult SWD collected were recorded by gender. All data from traps were analyzed to display mean weekly *D. suzukii* per trap for each of the regions. Mean daily temperatures for all seasons are presented together with trap catches.

### Degree-day estimation

Mean daily temperature data for all regions and seasons were converted to degree-day values relating development of SWD in cherries to indicate development for an early susceptible crop and to standardize thermal effects between seasons, production regions and microclimates. Degree-days were calculated using the single sine method with a lower threshold of 4°C and no upper threshold [44].

### Statistical analysis

In order to test the hypothesis that *D. suzukii* population predictions would correlate with trap counts, multiple regressions were performed on mean monthly trap captures from each region and the corresponding log-transformed population estimates using Statistica 7.1 [45].

No specific permissions were required for any of the field locations where data were collected. No field studies involved endangered or protected species. Because of the sensitive nature of *D. suzukii* presence, the corresponding author should be contacted if more information is required about specific field sites.

## Results

### Temperature data

**Figure 1:**
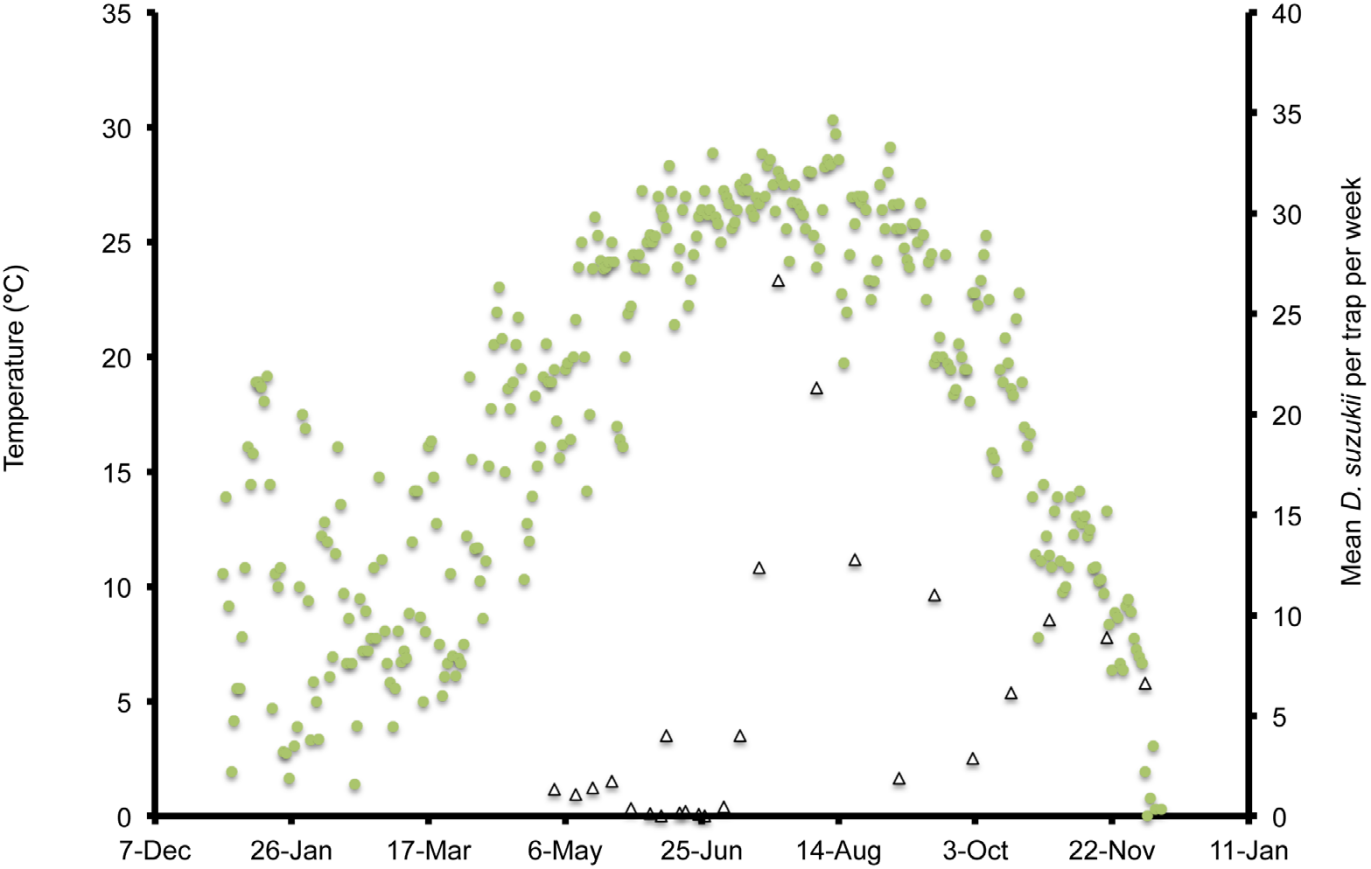
Mean daily temperatures and weekly *Drosophila suzukii* trap counts in Wilmington, North Carolina, US during 2013.

**Figure 2:**
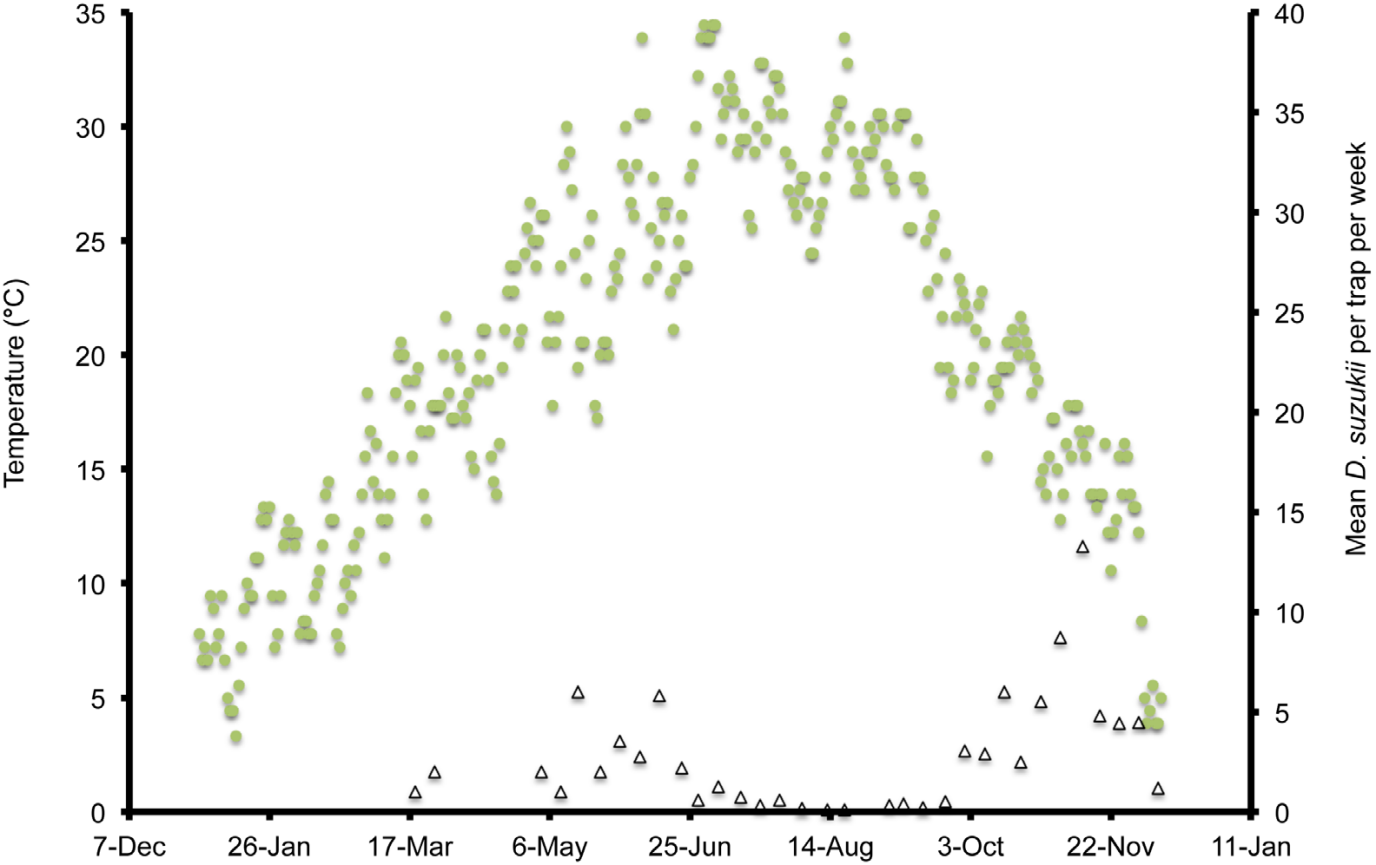
Mean daily temperatures and weekly *Drosophila suzukii* trap counts in Parlier, California, US during 2013.

**Figure 3:**
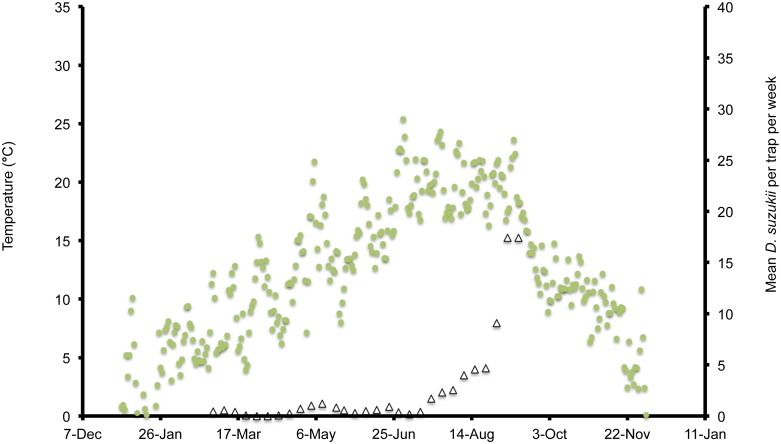
Mean daily temperatures and weekly *Drosophila suzukii* trap counts in Salem, Oregon, US during 2012.

**Figure 4:**
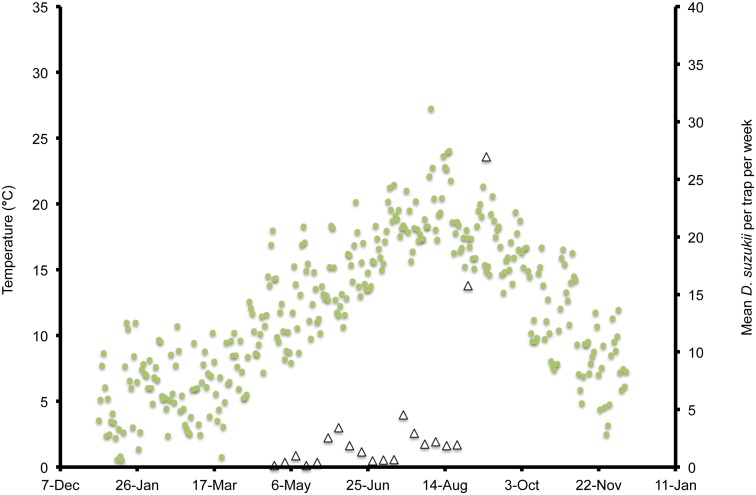
Mean daily temperatures and weekly *Drosophila suzukii* trap counts in Salem, Oregon, US during 2013.

In Wilmington, winter and late dormant mean temperatures were never below 0°C and reached 20°C multiple times prior to 31 April (Fig. 1). At this site, mean high summer temperatures exceeded 25°C multiple times during July, and mean low temperatures were below 10°C during November. In Parlier, winter and late dormant temperatures were never below 0°C and were higher than observed in both Salem and Wilmington during this period (Figs. 2, 3, 4). Here temperatures were above 25°C multiple times prior to 31 April. Mean daily temperatures were consistently above 25°C during June–September, and daily means in Parlier sometimes exceeded 33°C. In Parlier, temperatures dropped to below 10°C during November. In Salem, early-season temperatures were warmer during January and February of 2012, compared to 2013 (Figs. 3, 4). Temperatures observed from March through May were slightly higher during 2013 compared to 2012. In Salem, winter and late dormant temperatures were never below 0°C or above 15.3°C until 31 April 2013 (Fig. 4). Daily mean temperatures gradually increased to 25°C during July, after which daily mean temperatures dropped to below 10°C during November.

**Figure 5:**
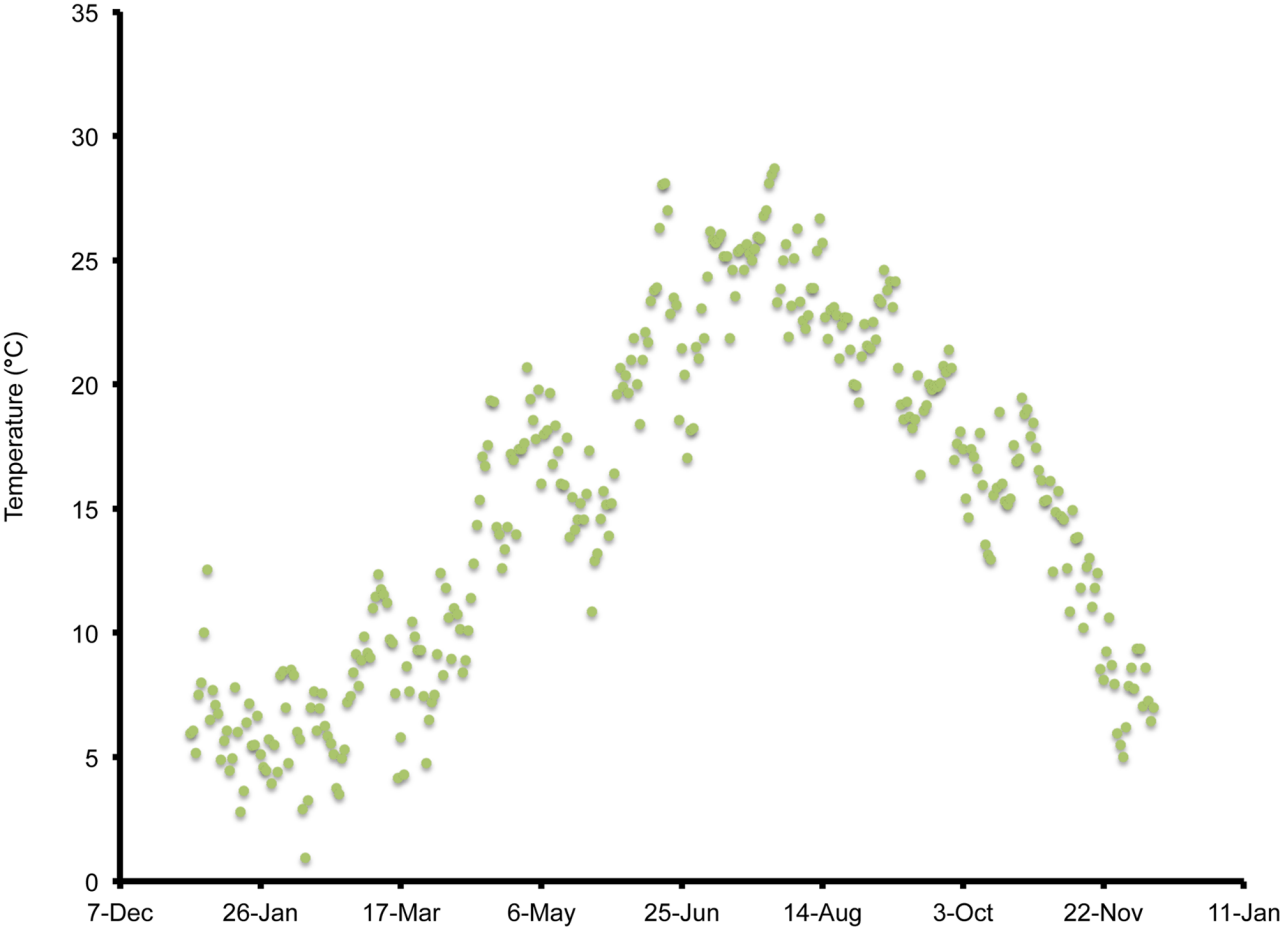
Mean daily temperatures in Riva del Garda, Trento Italy during 2013.

**Figure 6:**
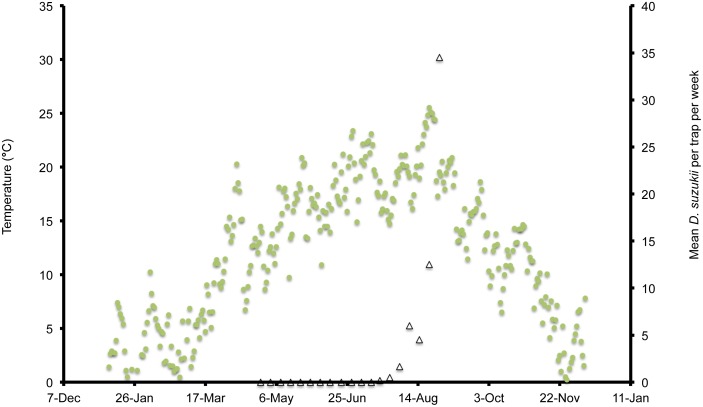
Mean daily temperatures and weekly *Drosophila suzukii* trap counts in Sant’Orsola, Trentino, Italy during 2013.

**Figure 7:**
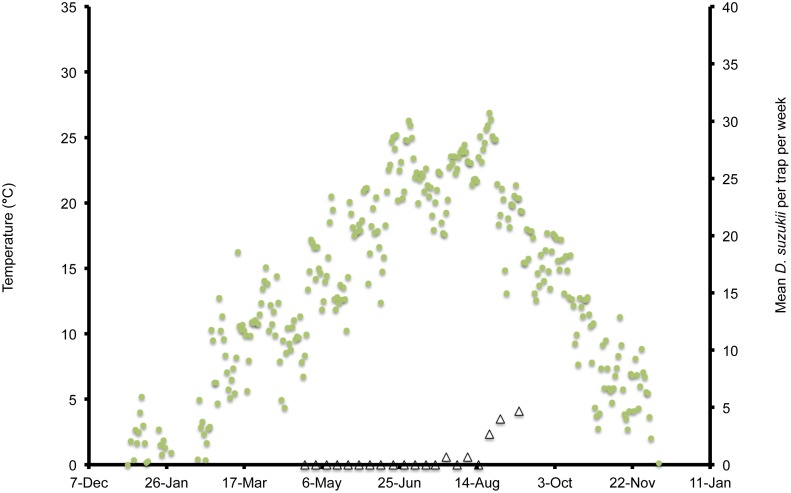
Mean daily temperatures and weekly *Drosophila suzukii* trap counts in Pergine, Trentino, Italy during 2012.

**Figure 8:**
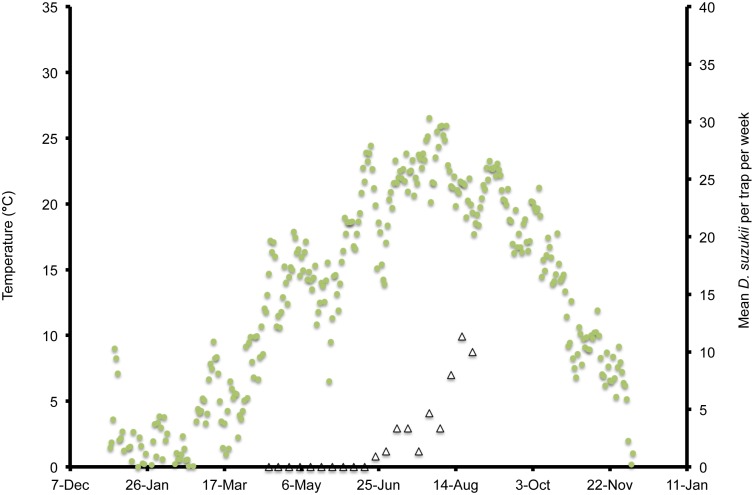
Mean daily temperatures and weekly *Drosophila suzukii* trap counts in Pergine, Trentino, Italy during 2013.

For the Italian sites Riva del Garda had higher late dormant and fall temperatures than Pergine and then by Sant’Orsola (Figs. 5, 6, 7, 8). Summer temperatures were similarly ranked higher in Riva del Garda, followed by Pergine and then Sant’Orsola during 2013. In Pergine during winter 2012 (Fig. 7), late dormant temperatures were frequently below 0°C and were as high as 20°C before 31 April. Fluctuations of temperature were more pronounced in 2012 in Pergine, compared to 2013 (Figs. 7, 8). Very low temperatures were recorded in Pergine from 3–12 February 2012, followed by relatively warm temperatures from 24 February to 13 March. Temperatures were also comparatively low from 9–11 April 2012. In Pergine, daily mean temperatures increased to 25°C during July, after which they dropped to below 10°C during November. Mean temperatures were well below 0°C during December.

### Population and stage-specific estimation

**Figure 9:**
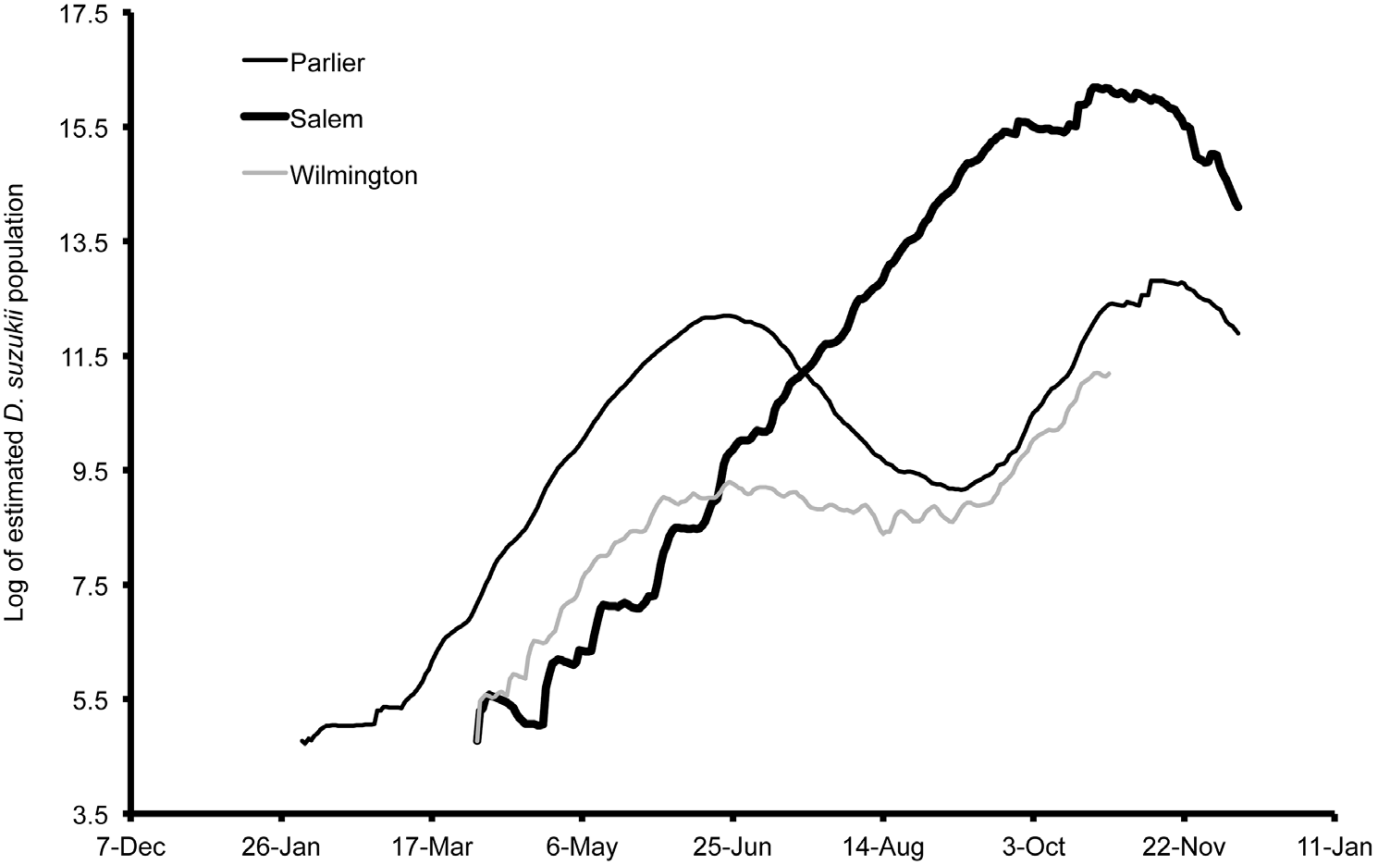
Log of estimated *Drosophila suzukii* populations for each of Parlier (California), Wilmington (North Carolina) and Salem (Oregon), US during 2013.

**Figure 10:**
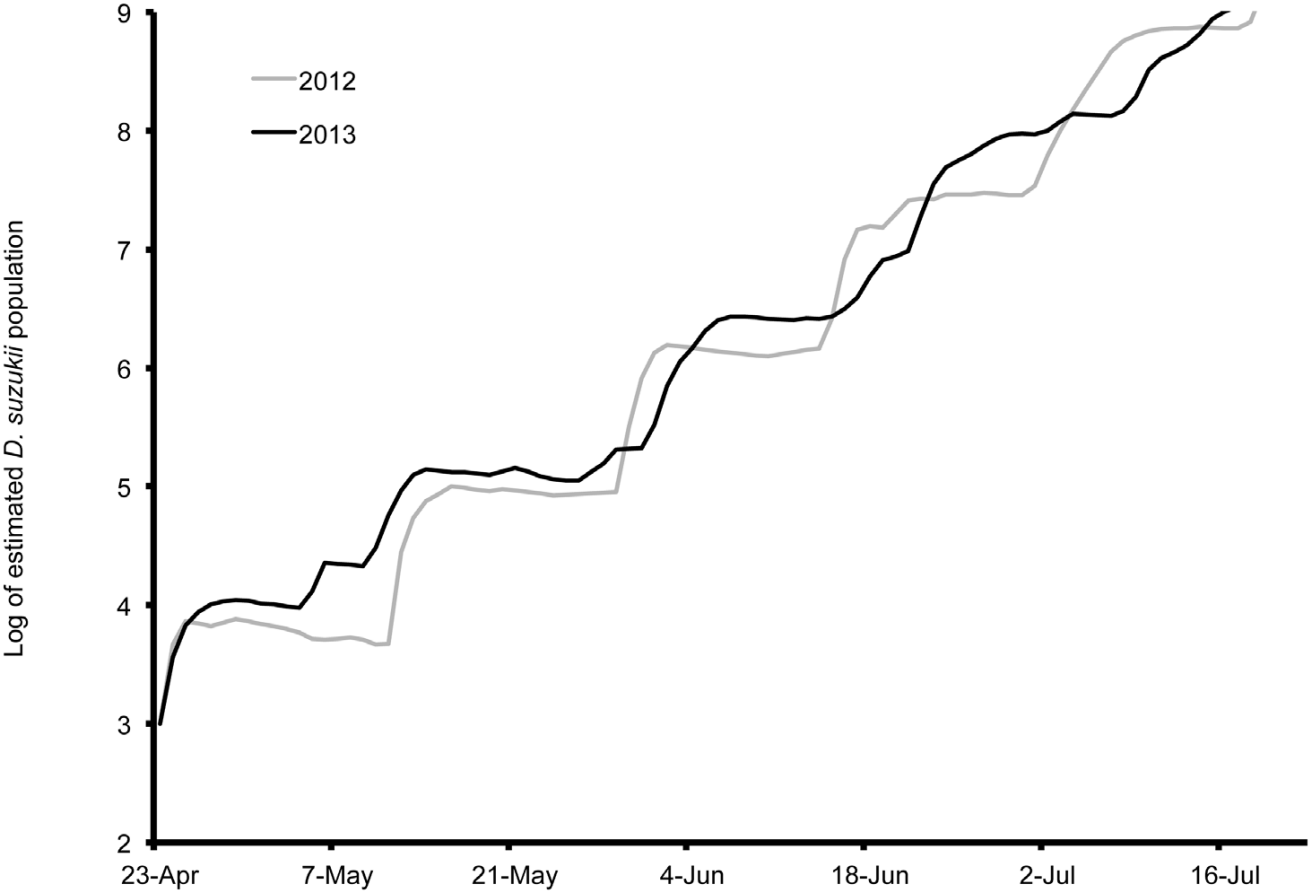
Log of estimated *Drosophila suzukii* populations for Salem (Oregon), US during 2012 and 2013.

**Figure 11:**
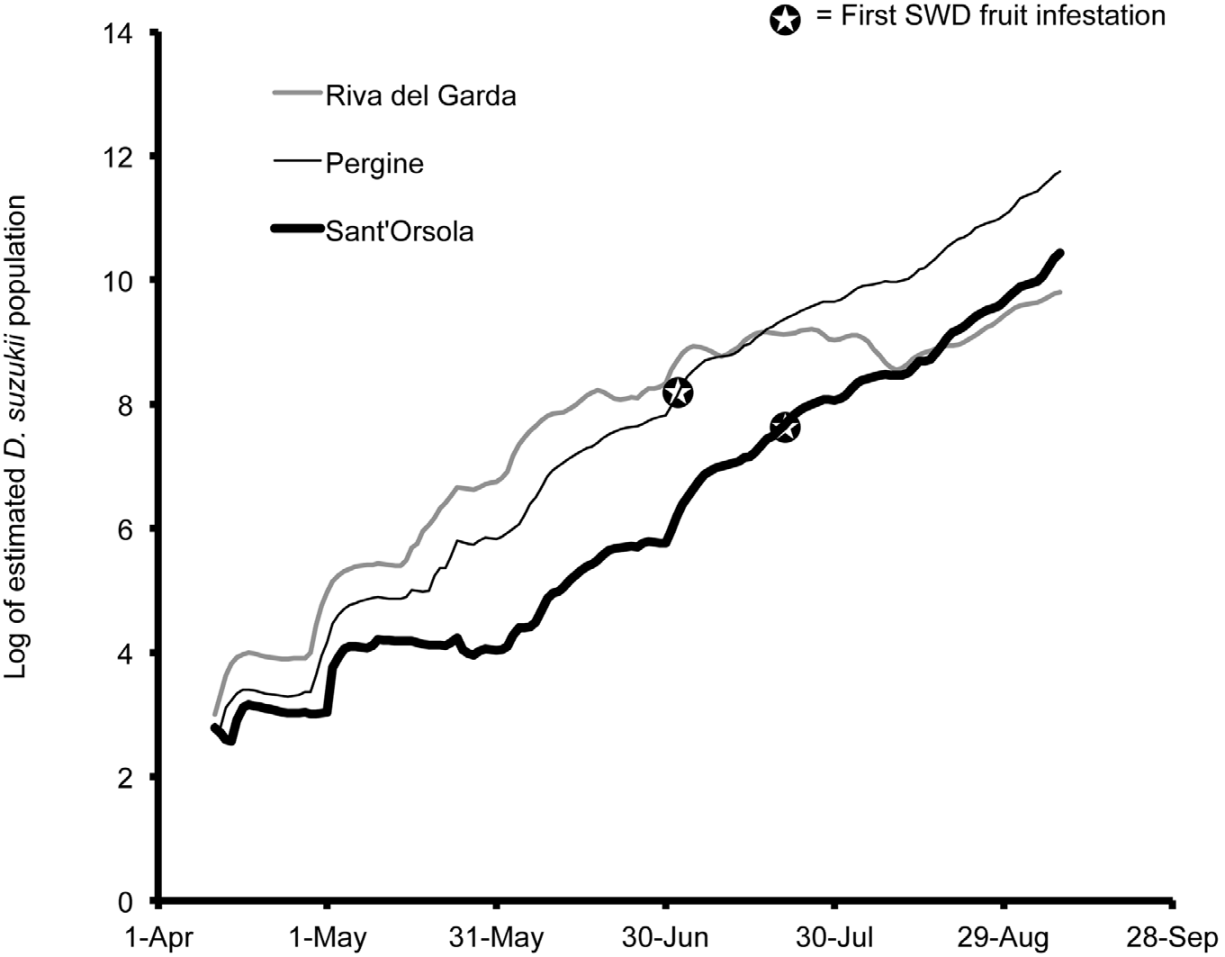
Log of estimated *Drosophila suzukii* populations for Riva del Garda, Pergine and Sant’Orsola, Italy during 2013.

**Figure 12:**
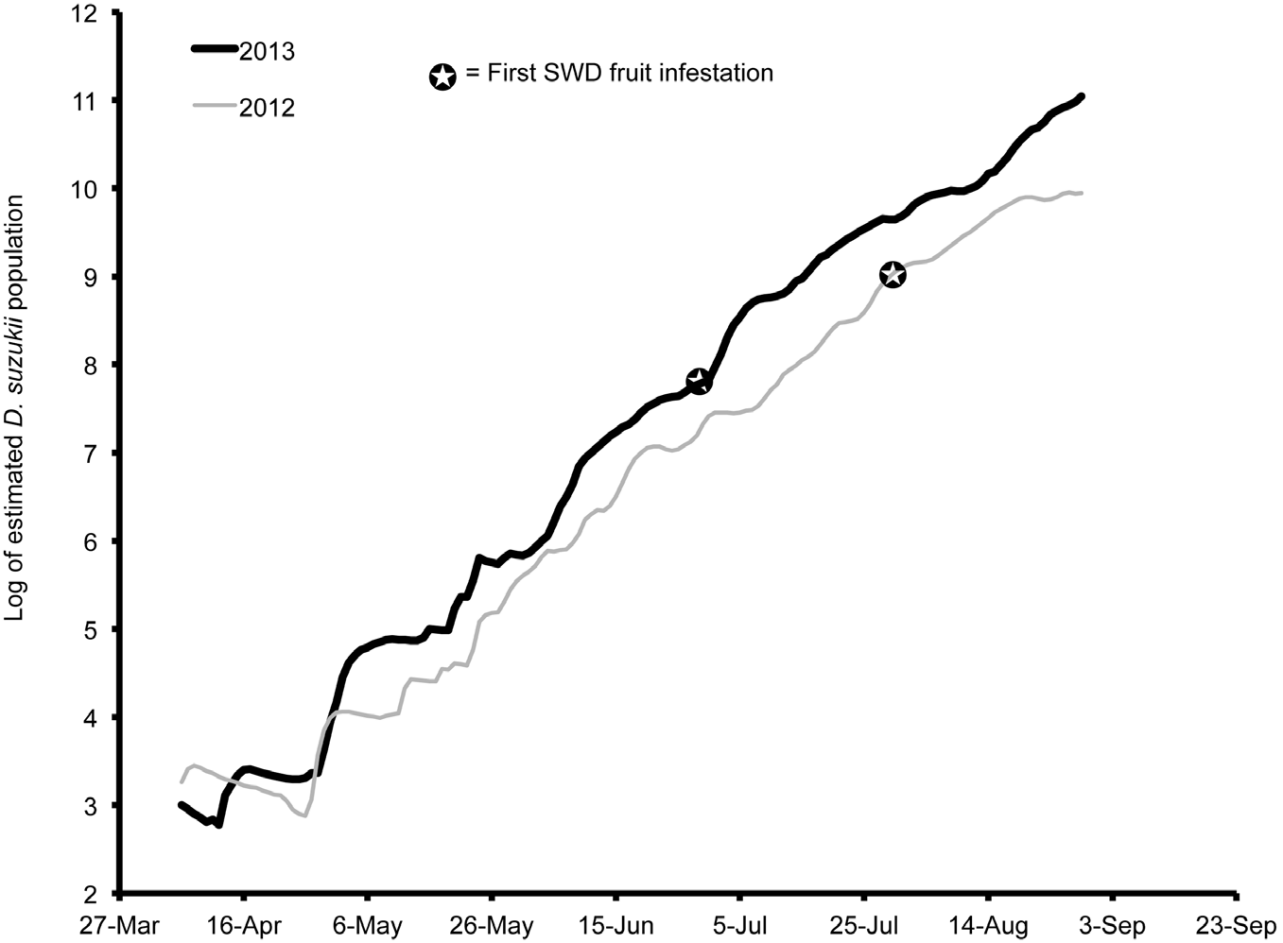
Log of estimated *Drosophila suzukii* populations for Pergine, Italy during 2012 and 2013.

Population estimates using temperature data indicate that *D. suzukii* populations are able to increase to high levels in all of the studied locations (Figs. 9, 10, 11, 12). The population estimates in all regions broadly tracked demographic trends of *D. suzukii* caught in traps (Table 1). When comparing early-season population estimates between Wilmington, Parlier, and Salem (Fig. 9), the population estimates were highest in 2013 in Wilmington followed by Parlier and then Salem. However, the population estimate for Salem surpassed Wilmington by 15 June and surpassed Parlier on 16 July, as Salem population estimates continued to climb while the latter sites experienced declining populations after reaching the first peak of their bimodal distributions. In Parlier, the early-season population peaked on 16 June, subsequently decreasing to a low on 10 September before increasing to a second population peak on 9 November, then decreasing again as winter progressed. In Wilmington, the population curve peaked on 21 June, then the population curve declined slightly for an extended period, followed by a second period of population increase beginning on 19 September to a population peak in November. In Salem, populations consistently increased from 25 April to a peak on 22 October, followed by a steep decrease. When comparing population estimates between seasons for the initial harvest period of early- to mid-season blueberries in Salem (19 May–19 July, Fig. 10), the majority of model outputs for this period estimated greater populations for 2013. When comparing populations along the elevation gradient of the three Italian sites, higher early-season populations were predicted at the lowest elevation Riva del Garda, followed by Pergine and then Sant’Orsola (Figs. 11). In Pergine, greater population numbers were estimated for the majority of the growing season during 2013 compared to 2012 (Fig. 12).

**Table 1:** Multiple regression correlation coefficients using for mean monthly *Drosophila suzukii* trap counts with log-transformed population estimates for 2012 and 2013.

Location	Adjusted R^2^ value	P value	d. f.	F value
Wilmington 2013	0.78	0.004	1, 4	23.2
Parlier 2013	0.72	0.009	1, 4	15.2
Salem 2012	0.58	0.001	1, 4	9.1
2013	0.8	0.039	1, 4	12.2
Pergine 2012	0.7	0.07	1, 4	7.1
2013	0.8	0.07	1, 4	7.6
Sant’Orsola 2013	0.65	0.06	1, 4	8.8

**Figure 13:**
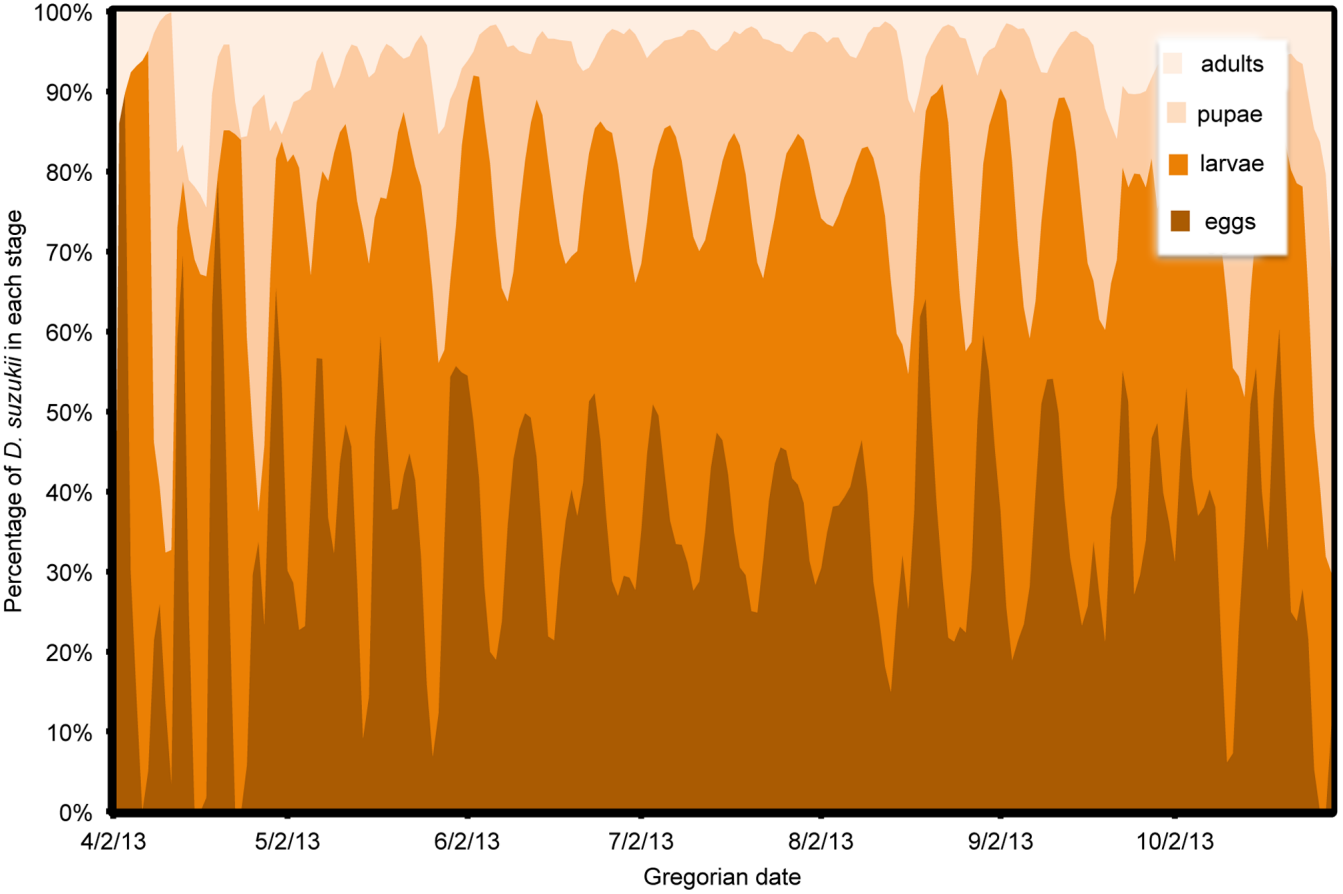
Estimates of *Drosophila suzukii* population structure in Wilmington (North Carolina), US during 2013.

**Figure 14:**
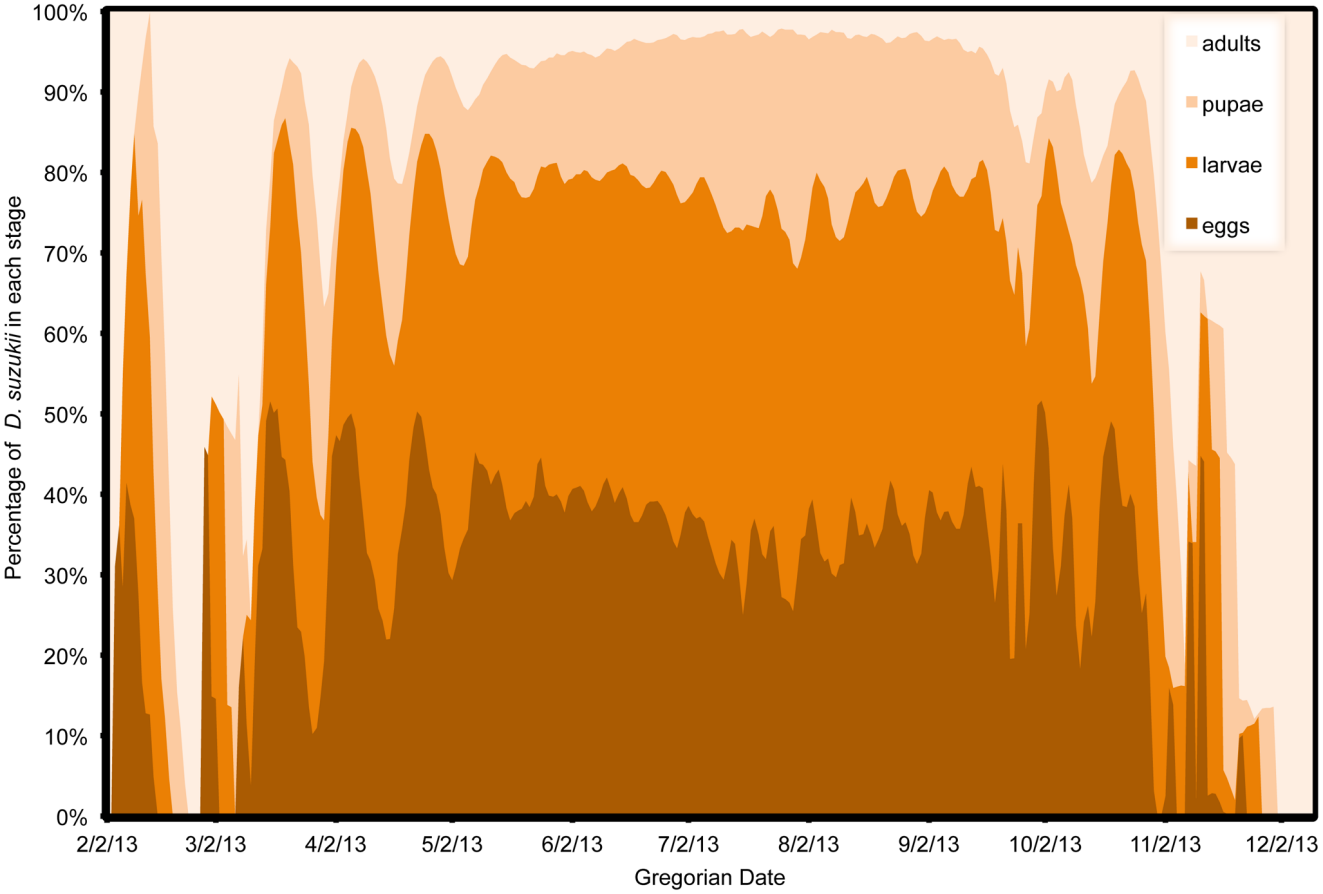
Estimates of *Drosophila suzukii* population structure in Parlier (California), US during 2013.

**Figure 15:**
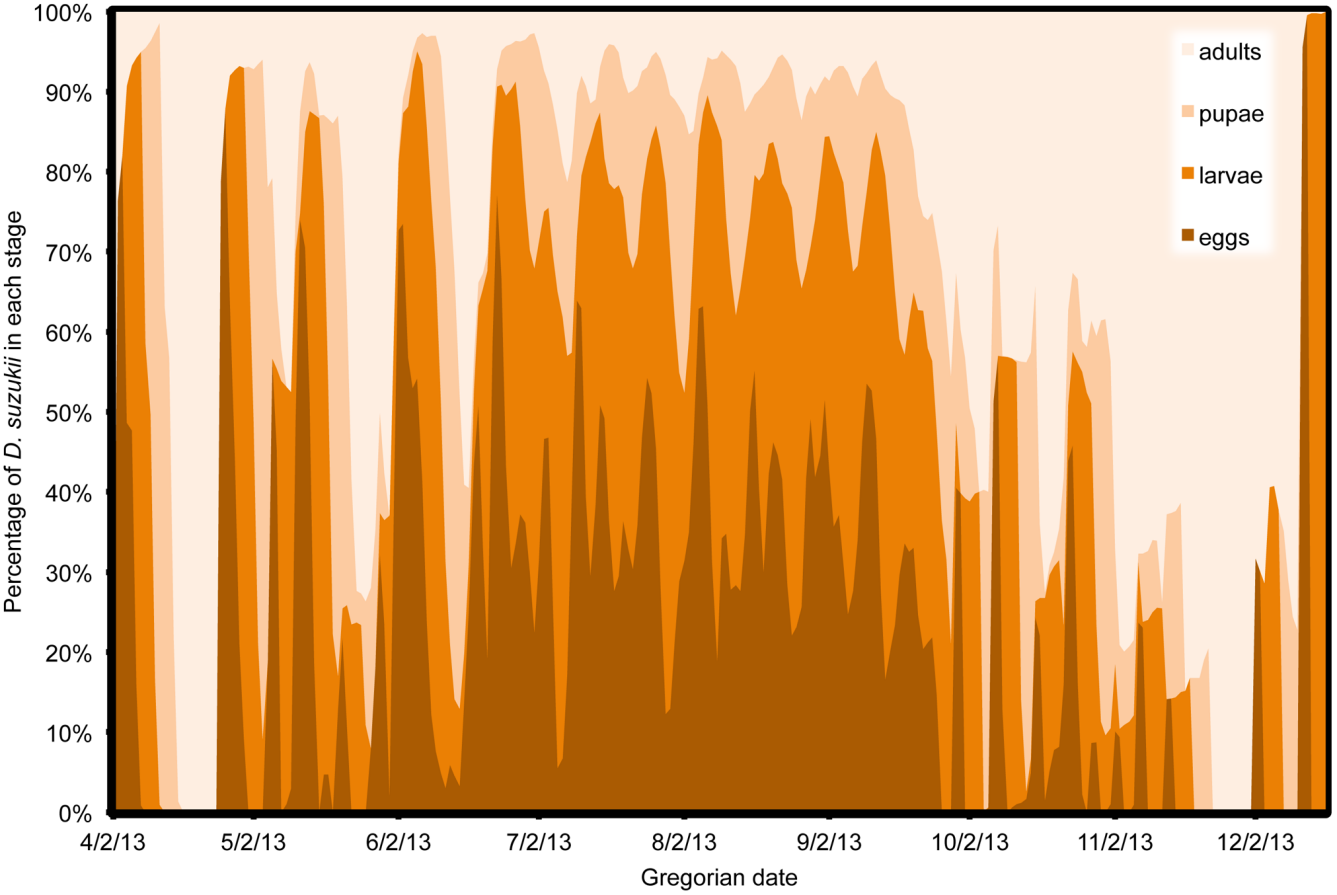
Estimates of *Drosophila suzukii* population structure in Salem (Oregon), US during 2013.

In all model predictions, immature life stages (eggs, larvae and pupae) comprised by far the majority of the population, except at the beginning or end of the season when adults tended to dominate (Figs. 13, 14, 15). One exception was Wilmington, where temperatures remained favorable for reproduction into the late fall so that immature stages remained a majority of the population (Fig. 13). In Salem, fall temperatures initially caused cessation of reproduction, leaving a majority of adults, but December temperatures allowed for some reproductive activity to occur (Fig. 14). In the early spring, a higher relative percentage of adults occurred due to the overwintering adults that were initiating their first reproduction. In part, this was an artifact of initiating the model with only older adult females. In the fall, environmental conditions became unfavorable for reproduction but may not have had strong effects on adult survival. Overall, no populations reached a completely stable age structure, but the highest relative stability for each site occurred in the middle of the season. Stability of age structure was the highest in Wilmington, followed by Parlier and finally Salem, which had a high degree of instability (Figs. 13, 14, 15). Demarcation of distinct generations was very clear for the first part of the season in Parlier (Fig. 14) and Salem (Fig. 15), but during the mid season at these sites and in Wilmington (Fig. 13), it was very difficult to distinguish individual generations to distinguish complete generations from partial generations.

### *Drosophila suzukii* trap counts

In Wilmington, *D. suzukii* counts were first recorded on 5 May 2013 at one fly per trap with an erratic increase to a peak in numbers at 26 flies per trap on 26 July (Fig. 1). After this period, the trap numbers gradually decreased to six flies per trap until 4 December, at which point *D. suzukii* trapping was discontinued. In Parlier, two population peaks were found during the crop season, one during the early part of the season, followed by a long mid-summer period without fly captures, and a second peak during the latter portion of the season (Fig. 2). Adult *D. suzukii* were first caught on 19 March 2013 at one fly per trap and increased to a high of six flies per trap on 16 May, after which they decreased to zero on 27 July. The trap numbers remained at this level until 19 September, after which numbers continued to increase into December. In Salem (2012, 2013) and Wilmington (2013) only one population peak was observed during the summer period (Fig. 1, 3, 4). During 2012 in Salem (Fig. 3), *D. suzukii* trap counts consistently increased starting on 5 July from one fly per trap per week to a maximum average of 17 flies per trap on 6 September. During 2013 in Salem (Fig. 4), the first *D. suzukii* trap counts were observed on 30 May at an average of three flies per trap per week and gradually increased until 10 September, when a maximum of 27 flies per trap was observed. The first trap counts during 2013 were therefore consistently recorded four weeks before those found in 2012 and higher levels of flies were found in traps during 2013 in Salem.

In Pergine and Sant’Orsola, fly counts were first observed 23 June 2013 (Figs. 6, 8), and on 7 July 2013 in respectively. In Italy, one population peak was visible each year for Pergine and Sant’Orsola. In 2012 in Pergine, the first flies were trapped on 25 July, approximately four weeks before those caught during 2013. The mean number of flies per trap per week increased to a peak of 4.6 on 10 September 2012 to a peak of 11.3 on 29 August 2013. Infested fruit was first found in 2013 on 24 June in Pergine (cherry), and on 19 July in Sant’Orsola (cherry, Fig. 11). First fruit infestation in Pergine in 2012 was determined on 24 June (cherry), compared to 28 July 2013 (cherry) (Fig. 12).

### Degree-day estimation

**Figure 16:**
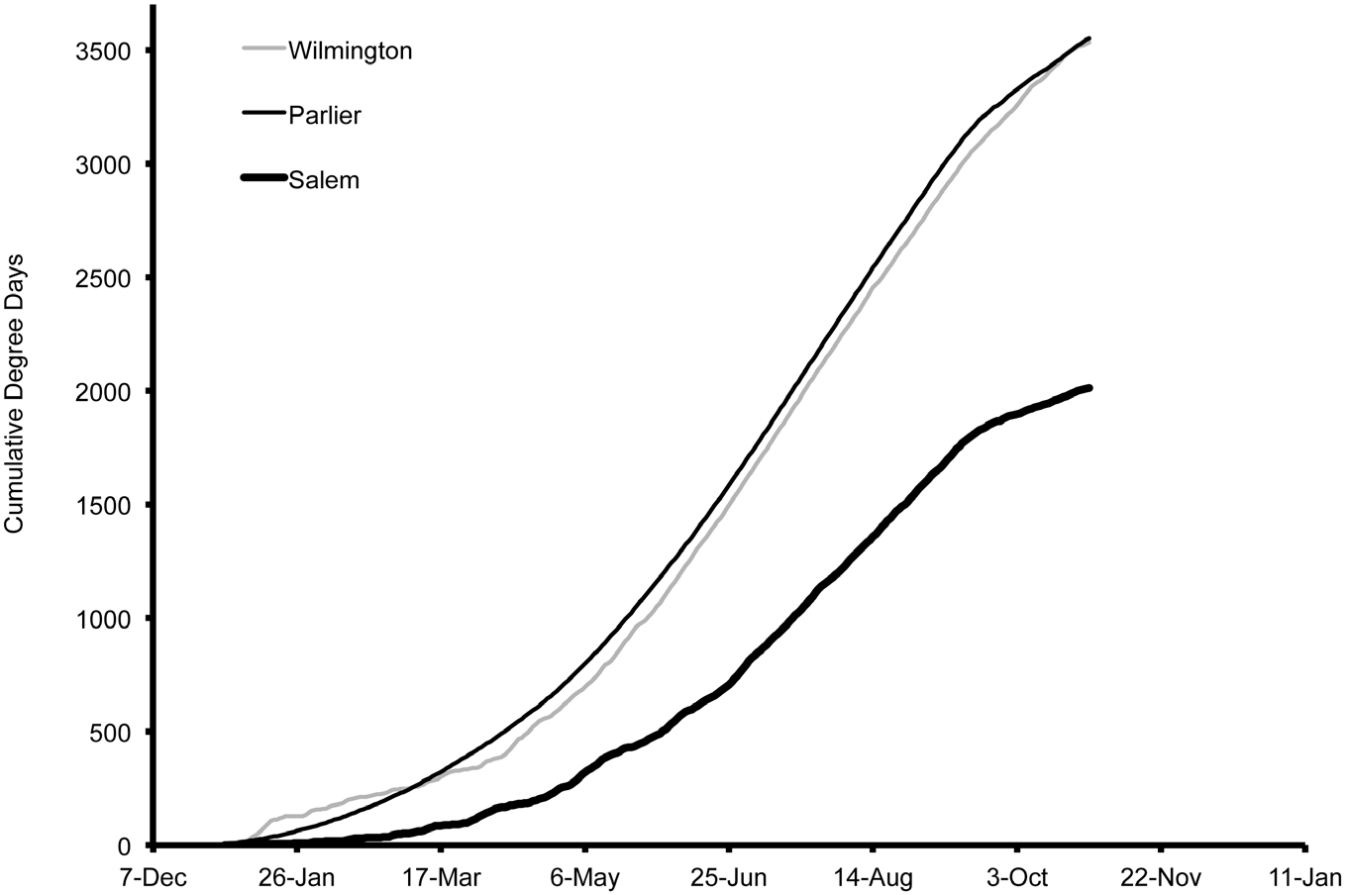
Degree-day estimations using 4°C as a lower threshold for Wilmington (North Carolina), Parlier (California), and Salem (Oregon), US during 2013.

**Figure 17:**
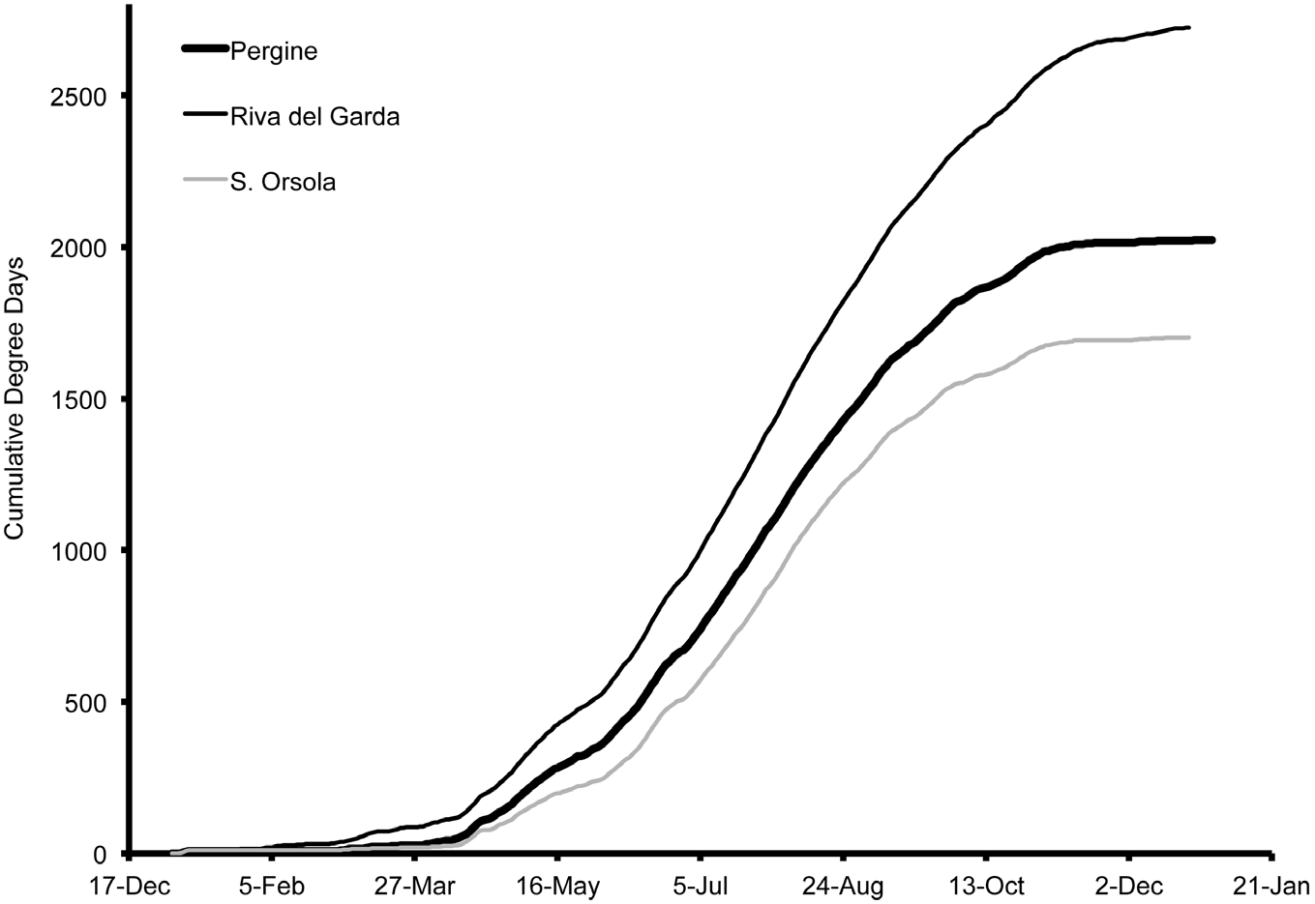
Degree-day estimations using 4°C as a lower threshold for Riva del Garda, Pergine and Sant’Orsola, Italy during 2013.

In order to compare environmental differences between all of the regions in this study, we illustrate degree-day accumulation for 2013 (Figs. 16, 17). When comparing differences between seasons, we found basic differences in accumulation for Salem and Pergine during 2012 and 2013. Of the three US locations, accumulation was initially the greatest in 2013 in Wilmington, followed by Parlier and then Salem (Fig. 16). However, accumulation of degree-days increased at a higher rate in Parlier and exceeded the accumulation in Wilmington by 3 March. The accumulation in Parlier was greatest for the remainder of the season and was closely followed by Wilmington. Salem accumulation was the least of the three regions presented in the US. In Salem, accumulation followed a similar pattern in 2012 compared to 2013, but the total number of degree-day degree-days was less in 2012 (DD = 1851) compared to 2013 (DD = 1988). In Italy during 2013, the accumulation was greatest in Riva del Garda, followed by Pergine, and then Sant’Orsola (Fig. 17). In Pergine, accumulation followed a broadly similar pattern in 2012 compared to 2013. Greater early-season degree-day accumulation in 2012 allowed a higher season-long total in 2012 (DD = 2112) compared to 2013 (DD = 1962).

## Discussion

In this study, we demonstrated how temperature-dependent fecundity and survival data could be integrated into a matrix population model to describe relative *D. suzukii* population increase and age structure according to environmental conditions in four environmentally-distinct fruit production regions. We found that the environment had major effects on how populations of *D. suzukii* behaved over a season in the different regions and that the population trends had implications for management. We also found that the different environments affected population stage-structure, and that stage structure also related to management of this pest. To see if independent measures would support population predictions, we used trap and fruit infestation data as well as degree-day estimates for comparison. In general, we found some corroboration of population trends with trap data, and to a limited extent with fruit infestation data. We found that degree-day accumulations did not reflect population predictions, and had limited capabilities to predict phenology or voltinism of this pest. The trap counts in our data are from either treated or untreated crops or crops that are unsuitable for *D. suzukii* population buildup. We realize that data from the traps placed in our study in some cases may, aside from other shortcomings, not provide an accurate early reflection of *D. suzukii* population levels.

The environment had important implications for when populations of *D. suzukii* were a threat for crops. When comparing predicted population trends from Wilmington, Parlier and Salem, it is apparent that in cooler regions such as Salem, early-ripening fruits would escape *D. suzukii* attack because early season temperatures are unsuitable for early population increase. In warmer regions such as Parlier and Wilmington, management of *D. suzukii* should begin as soon as susceptible fruits start to ripen, as favorability of early-season temperatures mean that populations of *D. suzukii* are high at the beginning of the season. In interior areas of California and lower elevations of Italy, there is a mid-season decrease in pest pressure as temperatures become very hot and less suitable to *D. suzukii*. This was true to a lesser extent in North Carolina and Riva del Garda in 2013, where mid-season populations declined only slightly. An implication of these predicted population trends is that management of *D. suzukii* during these periods could be less important relative to earlier and later periods when populations peak.

Clear differences in stage-specific population structure were found between Wilmington, Parlier and Salem. Temperatures appeared to be better suited for all life stage activities and survival in Wilmington throughout the calendar year, followed by Parlier and then Salem. Stability of population structure was highest in Wilmington, followed by Parlier and then Salem. The period characterized by the highest stability in population structure generally coincides with the period when fruit is harvested in some regions. This suggests that that there can be consistent pressure on the crop during harvest, which is a period when allowable pest management activity may be restricted. Stage-specific population structure was generally characterized by a small percentage of adults compared to immature stages in the *D. suzukii* population. This may explain why traps are such a poor indicator of fruit infestation. For pest management, the only life stage targeted currently is the adult stage. Unfortunately, the implications of the predicted age structure are that only a small percentage of the population is affected. This could help explain why frequent spray intervals are required to minimize crop damage from *D. suzukii*. Unless immature stages can be specifically targeted in future management, it will likely remain challenging to manage this pest with scheduled spray intervals in a way that breaks the life cycle of the insect.

While we were able to corroborate population projections with independent trap and fruit infestation data, we do not consider these data to be reliable or validating of early *D. suzukii* pest pressure [5], [14]. The level of precision for fruit infestation data from sampling is unknown. Previous literature indicates that action thresholds for fruit infestation demand fruit samples in far greater quantities of the numbers of fruit collected in the current study [50]. In order to get a 5% error rate for a sample containing 0.5% infested fruit, at least 600 fruits need to be collected and searched externally and internally through dissection. Given these limitations and the limited number of fruit that were collected in this study, fruit infestation could have happened earlier than observed. Erratic trap catches in Wilmington may have reflected yeast activity in monitoring traps used there. In the cases where we had trap and fruit infestation data, the first trap counts were observed during approximately the same period as when the first infested fruit was found. These findings illustrate the fact that traps cannot be seen as an early warning tool [5], [14].

Our predictions of stage structure in populations of *D. suzukii* illustrate the challenge of discerning distinct generations or predicting important life events in any useful way using a traditional degree-day model. Degree-day accumulations also did not directly reflect pest pressure as predicted by the population model, nor did the degree-day trajectories capture the subtlety in population fluctuations. When comparing estimations of population levels between years at Salem, there were clear differences in risk. Temperatures were more suitable for population buildup in Salem during the early portion of the season in 2013 compared to 2012. In Salem during May 2013, *D. suzukii* population projections were ten-fold higher compared to 2012, indicating the greater potential of crop losses for early-ripening crops. Assessing degree-day accumulations alone, differences between 2012 and 2013 were small and provided limited insight into the far higher early season risk in 2013 compared to 2012. For the annual comparison in Pergine, temperatures were within the optimal range for longer periods during 2013 compared to 2012, and *D. suzukii* population pressure was projected to be higher. In Pergine, the differences in population are not clearly reflected by degree-day estimations, because lower degree-day accumulation was measured in 2013 compared to 2012.

For all production regions, *D. suzukii* population estimation has application for use as a virtual laboratory where ‘what-if’ statements can be raised and answered prior to management action, by simulating population changes as typically achieved during pesticide intervention, *Wolbachia* infection [46], [47], or biological control. Age-structured population models can be used to simulate mortality on specific life stages to predict how different management strategies affect *D. suzukii* pressure. These strategies can then be validated by field implementation. Population estimation in this study was not aimed at simulating the behavior of individuals or populations of *D. suzukii* as found in other more complex models for insects of medical importance [20], [24]–[26], [48]. Winter survival, availability of suitable host medium, nutrient sources, humidity and suitable host plant environments were not taken into consideration in this study. These factors can have strong effects population densities. It is clear that behavior of *D. suzukii* is important [48]. The migration of flies to track favorable environmental conditions and host suitability was also not taken into consideration when making these estimates [49]. The mechanisms of *D. suzukii* thermal extreme tolerance [30] are not well documented and need further investigation in order to determine potential adaptation or behavioral mitigation to temperature extremes. Existing literature on *Drosophila* indicates that mechanisms of thermal tolerance may be influenced by the gene expression of heat shock proteins [50]. These mechanisms have not been studied in *D. suzukii* but data from future studies on the influence of these factors would benefit population models such as presented here. Other refinements of our model could potentially account for metapopulation dynamics, host availability, and overwintering survival, and density dependence.

Like all models, ours necessarily makes assumptions and presents a simplified representation of complex ecosystems, ignoring some factors that may influence *D. suzukii* population levels. However, temperature is clearly one of the most important factors for *D. suzukii* population growth. This model has clear application for predicting relative pressure from *D. suzukii* in crops, and can be used as a temperature-related and physiology-based comparative risk tool for pending larval infestation. Further application of the population projections would be to extend them into the future based on weather forecasts. Validation of this model will require controlled experiments on *D. suzukii* to test hypotheses about survival and fecundity based on model output in response to environmental conditions. For example, caged populations of a known size could be subjected to temperature simulations over intervals of time to assess population trajectories and age structures for comparison with model predictions. Additional validation studies could also include passive or live traps to capture flies over the season to be reared in field cages for assessment of survival and reproductions.

We believe that the model described here is useful to approximate population levels, to more clearly define the age structure of populations, and to provide additional information that may aid in decision-making for *D. suzukii*. Given the many complexities in predicting populations, we argue that absolute precision is not necessary to identify effective management interventions or to improve understanding of this pest. We recognize the limitations of our projection model but believe that it represents a novel technique and a potentially powerful tool for management and research on *D. suzukii* and other damaging insects.
